# Mechanical Stability of Screw-Retained Monolithic and Bi-layer Posterior Hybrid Abutment Crowns after Thermomechanical Loading: An In Vitro Study

**DOI:** 10.3390/ma14247539

**Published:** 2021-12-08

**Authors:** Frank A. Spitznagel, Estevam A. Bonfante, Tiago M. B. Campos, Maximilian A. Vollmer, Johannes Boldt, Sam Doerken, Petra C. Gierthmuehlen

**Affiliations:** 1 Department of Prosthodontics, Medical Faculty and University Hospital Düsseldorf, Heinrich Heine University, Moorenstraße 5, 40225 Düsseldorf, Germany; info@zahnarzt-im-bunker.de (J.B.); petra.gierthmuehlen@med.uni-duesseldorf.de (P.C.G.); 2 Department of Prosthodontics and Periodontology, Bauru School of Dentistry, University of Sao Paulo, Bauru 17000-000, Brazil; estevam.bonfante@fob.usp.br (E.A.B.); moreiratiago22@gmail.com (T.M.B.C.); 3 Private Practice, 88079 Tettnang, Germany; info@zahnarzt-in-tettnang.de; 4 Private Practice, 47807 Krefeld, Germany; 5 Institute of Medical Biometry and Statistics, Faculty of Medicine and Medical Center, University of Freiburg, 79108 Freiburg im Breisgau, Germany; sam.doerken@datamap.de

**Keywords:** ceramics, dental implants, fatigue, implant supported dental prosthesis, titanium bases

## Abstract

To evaluate the failure-load and survival-rate of screw-retained monolithic and bi-layered crowns bonded to titanium-bases before and after mouth-motion fatigue, 72 titanium-implants (SICvantage-max, SIC-invent-AG) were restored with three groups (*n* = 24) of screw-retained CAD/CAM implant-supported-single-crowns (ISSC) bonded to titanium-bases: porcelain-fused-to-metal (PFM-control), porcelain-fused-to-zirconia (PFZ-test) and monolithic LDS (LDS-test). Half of the specimens (*n* = 12/group) were subjected to fatigue in a chewing-simulator (1.2 million cycles, 198 N, 1.67 Hz, thermocycling 5–55 °C). All samples were exposed to single-load-to-failure without (PFM0, PFZ0, LDS0) or with fatigue (PFM1, PFZ1, LDS1). Comparisons were statistically analyzed with t-tests and regression-models and corrected for multiple-testing using the Student–Neuman–Keuls method. All PFM and LDS crowns survived fatigue exposure, whereas 16.7% of PFZ showed chipping failures. The mean failure-loads (±SD) were: PFM0: 2633 ± 389 N, PFM1: 2349 ± 578 N, PFZ0: 2152 ± 572 N, PFZ1: 1686 ± 691 N, LDS0: 2981 ± 798 N, LDS1: 2722 ± 497 N. Fatigue did not influence load to failure of any group. PFZ ISSC showed significantly lower failure-loads than monolithic-LDS regardless of artificial aging (*p* < 0.05). PFM ISSC showed significantly higher failure loads after fatigue than PFZ (*p* = 0.032). All ISSC failed in a range above physiological chewing forces. Premature chipping fractures might occur in PFZ ISSC. Monolithic-LDS ISSC showed high reliability as an all-ceramic material for screw-retained posterior hybrid-abutment-crowns.

## 1. Introduction

Implant placement and subsequent restoration with an implant-supported single crown (ISSC) is a well-established treatment option to replace a single missing tooth [1,2]. Porcelain-fused-to-metal (PFM) implant crowns are still considered as the gold-standard with an estimated survival rate of 98.3% after 5 years [3]. Yet, all-ceramic ISSC are gaining market share due to their esthetic and tooth-like appearance with high survival rates of 93–97.6% [2,3] and 94.4% [2] after 5 and 10 years of observation, respectively. However, both PFM and all-ceramic ISSC are prone to technical complications, with fractures of the veneering ceramic being the most frequent ones. Current systematic reviews reported chipping incidence rates of 2.8–9% for all-ceramic [2,3] and 2.9% for metal-ceramic [3] ISSC after 5 years. Moreover, porcelain-fused-to-zirconia (PFZ) ISSCs appear even more susceptible to chipping events than PFM implant crowns [3]. As a consequence, monolithic all-ceramic reconstructions evolved to avoid the technique sensitive veneering process [4]. Advances in CAD/CAM dentistry and the implementation of digital technologies favor the application of monolithic all-ceramic ISSC [5,6,7]. In addition, CAD/CAM manufactured monolithic posterior ISSC in a complete digital workflow resulted in more time efficiency and effectiveness compared to conventionally produced implant crowns [6,7,8]. Especially, prefabricated monolithic lithium disilicate (LDS) blanks bonded to an adhesive titanium base (hybrid abutment crowns) seem to be a cost- and time-efficient treatment option for the posterior dentition [5,9]. Furthermore, CAD/CAM-fabricated monolithic LDS crowns could reduce treatment costs by more than 30% due to a shorter manufacturing time, in contrast to CAD/CAM produced PFZ crowns [6]. The digital workflow is preferable not only from an economic standpoint, in terms of clinical efficiency and impression time, but it also yielded in higher patient acceptance [10]. An RCT analyzed the need of clinical adjustments and the precision of posterior monolithic LDS hybrid abutment crowns compared to PFZ implant crowns [11]. Chairside produced monolithic LDS CAD/CAM ISSC required fewer adjustments and provided more accurate results [11]. Preliminary survival rates of 100% have been reported in clinical studies with press- and CAD/CAM-fabricated posterior monolithic LDS hybrid abutment crowns over 1 to 3 years of follow-up [12,13,14]. However, clinical long-term data and robust prosthetic treatment concepts are still missing [15].

To be recognized as an equal reliable or even superior prosthetic treatment option, the comparison of monolithic LDS ISSC to the PFM gold-standard and to widely used PFZ implant crowns is needed. Therefore, the aim of this laboratory study was to investigate and compare monolithic screw-retained hybrid abutment LDS crowns and bi-layer screw-retained PFM and PFZ ISSC with regard to their in vitro survival rate over a simulated 5-year period and their load to failure. The following null hypotheses were formulated: (i) type of material (LDS vs. PFZ vs. PFM) and (ii) fatigue application do not influence the failure load of posterior hybrid abutment crowns.

## 2. Materials and Methods

**Figure 1:**
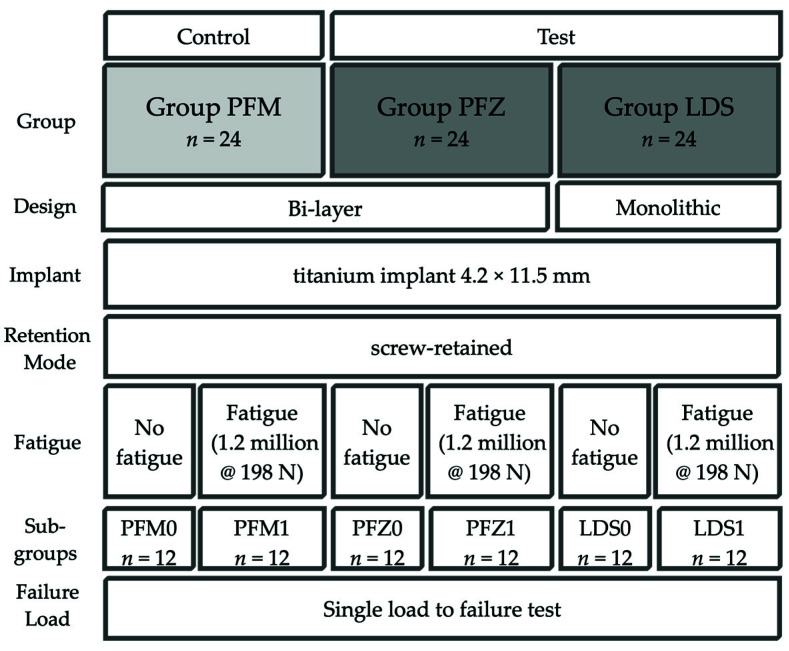
Study setup.

In this laboratory study, titanium implants (SICvantage max, SIC invent AG, Basel, Switzerland) with an internal conical connection and a platform switch were used as test specimens. Seventy-two implant samples of 4.2 mm in diameter and 11.5 mm in length were connected with screw-retained ISSC bonded to a prefabricated titanium base (SICvantage CAD/CAM Abutment red, SIC invent AG, gingival height 1 mm, prosthetic height 4.7 mm) and divided into two test groups and one control group of 24 specimens each according to the type of material (Figure 1):

- Control Group PFM: bi-layer porcelain-fused-to-metal crown (Ivoclar non-precious metal 4All/IPS Inline PoM, both Ivoclar Vivadent, Schaan, Liechtenstein);
- Test Group PFZ: bi-layer porcelain-pressed-to-zirconia crown (Incoris ZI meso, Dentsply Sirona and IPS e.max ZirPress, Ivoclar Vivadent);
- Test Group LDS: monolithic LDS (IPS e.max CAD Abutment solutions, Ivoclar Vivadent).

### 2.1. Fabrication of Crowns

For standardization, one implant was embedded in a master model (Frasaco-Model, Frasaco, Tettnang, Germany) in the position of a mandibular first molar. The prosthetically correct position for a screw-retained restoration was selected. The master model was scanned (InEos X5, Dentsply Sirona, Charlotte, NC, USA) and a master design of a mandibular molar crown (InLab 15.1, Dentsply Sirona, Charlotte, NC, USA) was used for all crowns in order to produce identical and comparable test specimens. All ISSC were produced in a commercial dental laboratory by the same master dental technician following strictly the manufacturer’s recommendations.

#### 2.1.1. Group LDS

ISSC of Group LDS were milled from IPS e.max CAD Abutment solutions LT A2 in a five-axis milling machine (inLab MC X5, Dentsply Sirona, Charlotte, NC, USA) followed by final crystallization/glaze firing and polishing.

#### 2.1.2. Group PFZ

The master crown design for Group PFZ and PFM was split to generate a separate framework and veneer layer. The zirconia substructure and the veneer layer of Group PFZ were milled (inLab MC X5, Dentsply Sirona, Charlotte, NC, USA) out of a prefabricated zirconia blank (InCoris ZI meso S F 0.5, Dentsply Sirona, Charlotte, NC, USA) and a wax blank (ProArt Wax Blue, Ivoclar Vivadent). The zirconia substructure was then sintered in a furnace (InFire HTC, Dentsply Sirona, Charlotte, NC, USA) and a zirliner firing (IPS e.max Ceram ZirLiner 2, Ivoclar Vivadent) was conducted. Subsequently, the zirconia substructure with wax veneer was embedded (IPS Press Vest Speed, Ivoclar Vivadent) and the overpress technique was applied (IPS e.max ZirPress Shade HT A2, Programat EP 5010 furnace, both Ivoclar Vivadent). Glaze firing (IPS e.max Ceram Glaze, Ivoclar Vivadent) and polishing was performed afterwards.

#### 2.1.3. Group PFM

Group PFM was fabricated accordingly, the metal substructure and veneer layer were designed and then milled out of a wax blank (ProArt Wax Blue, Ivoclar Vivadent). The metal framework was then casted from non-precious alloy (4all, Ivoclar Vivadent). Subsequently, an opaquer firing (IPS InLine paste opaquer, Ivoclar Vivadent) was conducted and the framework with the wax veneer was embedded (IPS Press Vest Speed, Ivoclar Vivadent) and pressed (InLine PoM 3, Programat EP 5010 furnace, both Ivoclar Vivadent). Afterwards, glaze firing (IPS InLine Glaze, Ivoclar Vivadent) and polishing were performed.

### 2.2. Preparation of Specimens

An autopolymerizing polyester resin (Technovit 4000, Kulzer, Hanau, Germany), with a modulus of elasticity of nearly 12 GPa, was used as an embedding material. All titanium implants were covered up to the first thread. The resin simulates the elastic reaction of the surrounding bone tissue during loading [16,17,18].

ISSC of Group LDS were pretreated with 4.9% hydrofluoric acid (IPS ceramic etching gel, Ivoclar Vivadent) for 20 s, washed with water, dried with oil-free air stream followed by application of a silane (Monobond Plus, Ivoclar Vivadent). The inner surface of Group PFZ and PFM were air-particle abraded with 110 μm aluminum-oxide at a pressure of 2 bar. The surface of the titanium bases (SICvantage CAD/CAM Abutment red, SIC Invent AG, Basel, Switzerland), with a prosthetic height of 4.7 mm were first mechanically pretreated via sandblasting with 50 μm aluminum-oxide (2 bar pressure) and afterwards chemically modified (Monobond Plus).

After steam-cleaning, all ISSC were resin-bonded with a composite (Multilink Hybrid Abutment, Ivoclar Vivadent) to the titanium base. The hybrid abutment crowns were tightened with 20 Ncm using a torque control and retightened after 10 min to avoid screw loosening [19,20]. The screw access holes were closed with teflon tape (Kirchhoff GmbH, Wallenhorst, Germany) and a composite filling material (Tetric EvoCeram Bulk Fill, Ivoclar Vivadent).

### 2.3. Cyclic Loading and Single Load to Failure (SLF)

Twelve samples of each group (LDS1, PFZ1, PFM1, Figure 1) were aged in a mouth-motion fatigue simulator (1.2 million cycles, 198 N, 1.6 Hz, CS-4.8 professional line, SD Mechatronik, Feldkirchen-Westerham, Germany) and simultaneously thermocycled (5 °C to 55 °C, dwell time 120 s) equivalent to five years of clinical service [21,22]. Cyclic fatigue testing was performed by sliding a steatite indenter (r = 3 mm, Hoechst CeramTec, Wunsiedel, Germany) down the mesiolingual cusp towards the central fossa of the restoration (horizontal movement of 0.5 mm) simulating aspects of natural chewing [23]. Specimens were vertically positioned and loaded without angulation. Steatite balls with a diameter of 6 mm, equalizing a cusp of an antagonist molar, served as standardized intenders [24]. During thermomechanical loading, the samples were examined twice a day for cracks and fractures of the ISSC. After fatigue testing, all samples were vertically loaded until failure in a universal testing machine (Zwick Z010/TN2S, Zwick Roell, Ulm, Germany). The force was applied at the same contact point as during cyclic loading with a vertical speed of 1.5 mm/min. A steel ball with the same diameter of 6 mm was chosen as a load indenter. Fractures of the veneering ceramic (cracks, chipping), catastrophic core fractures as well as implant or screw fractures were defined as failure.

### 2.4. Failure Analysis

Failed samples were first analyzed in a polarized light microscope (AxioZoom V.16, Zeiss, Oberkochen, Germany) and most representative specimens were further subjected to qualitative fractographic analyses via scanning electron microscope (Vega 3, Tescan, Kohoutovice, Czech Republic) to determine the mode of failure.

### 2.5. Statistical Analysis

Statistical Analysis was performed with STATA 14 (StataCorp LLC, College Station, TX, USA). Effects of the chewing simulator on failure load were analyzed using the t-test. The assumptions of normality of the data and equality of variance between comparison groups were not formally tested due to limited sample size but were deemed adequate from an inspection of the quartile distributions. Pairwise group comparisons were performed in a regression model, using material group (LDS, PFM, PFZ) and exposure to mouth-motion fatigue simulator as covariates. To account for multiple testing, the Student–Newman–Keuls method was applied to determine adjusted *p*-values. The level of significance was set at *p* < 0.05. An initial sample size calculation revealed a minimum sample size of 10 to obtain a power of 80% with an effect size of 0.65 (G*Power 3.1, HHU-University, Düsseldorf, Germany). To account for possible drop-outs, 12 specimens per group were therefore evaluated.

## 3. Results

### 3.1. Cyclic Loading

**Figure 2:**
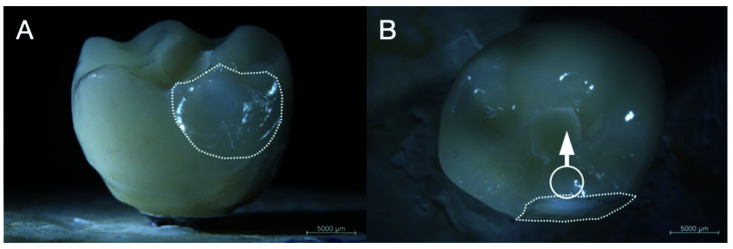
Sample no. 7 of group PFZ1 with pronounced chipping extending mesiolingually. (**A**) labial and (**B**) occlusal overview. Circle and arrow indicate chewing simulation path; dotted line indicates chipping area.

**Figure 3:**
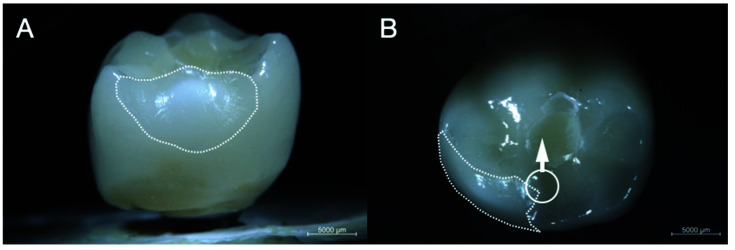
Sample no. 8 of group PFZ1 with pronounced chipping extending distolingually. (**A**) labial and (**B**) occlusal overview. Circle and arrow indicate chewing simulation path; dotted line indicates chipping area.

No fractures, cracks or screw loosening occurred on implants, abutments and ISSC of group LDS1 and PFM1 resulting in a survival rate of 100% after chewing simulation. Two ISSC (16.67%) of group PFZ1 showed extended chip fractures during fatigue and were, therefore, excluded from further analysis. Hence, specimens of group PFZ1 yielded in a survival rate of 83.3% after fatigue. The location of the chipping was found on the lingual side of both restorations. One sample showed an adhesive fracture with exposure of the underlying zirconia framework (Figure 2) after 664,000 cycles and one specimen revealed a cohesive fracture after 145,000 cycles, leaving a thin veneering layer over the framework ceramic (Figure 3).

### 3.2. Single-Load-to Failure

**Figure 4:**
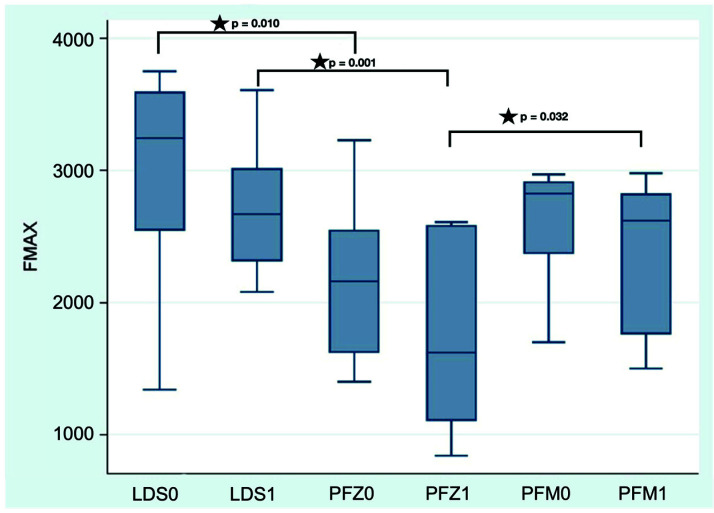
Boxplot of failure load (F_max_ in N). Asterisk indicates statistically significance (0 = groups without fatigue, 1 = groups with fatigue).

Monolithic LDS ISSCs achieved the highest numeric failure loads both before and after fatigue (Table 1, Figure 4). Within the bi-layer restorations, group PFZ showed lower values than PFM irrespective of fatigue (Table 1). Comparisons of failure load showed no significant results for all groups before and after fatigue (Table 1). For comparisons between different groups, adjusted *p*-values could be computed. Significantly lower failure loads could be detected after dynamic loading for PFZ1 compared to PFM1 (*p* = 0.032). No differences between PFM and PFZ could be detected before fatigue (*p* = 0.129). Irrespective of fatigue application, monolithic LDS crowns showed significantly higher failure loads than bi-layer PFZ (*p* < 0.01). No differences between LDS and PFM could be observed irrespective of fatigue exposure (*p* > 0.05).

### 3.3. Fractographic Analysis

**Figure 5:**
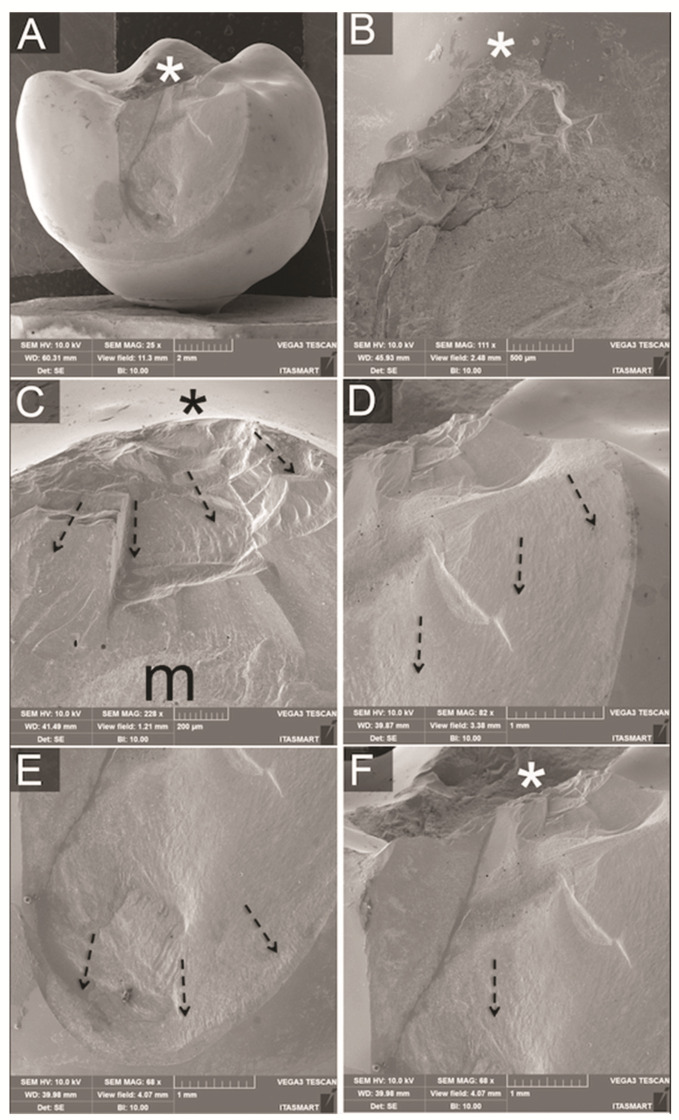
(**A**) Buccal view SEM micrograph of a fractured PFM crown showing porcelain veneer fracture extension and indentation area (asterisk). (**B**) Occlusal view shows indentation area (asterisk) originating the fracture. (**C**) Magnification of indentation area shows quasiplastic damage right below indentation area and hackle lines (dotted arrows) indicating direction of crack propagation. Exposure of the metallic framework (m) was evidenced in a small area. Figures (**D**–**F**) are clockwise magnifications of a fracture margin magnified in a buccal perspective, showing hackle lines (dotted arrows) indicating the direction of crack propagation from indentation towards fracture margins.

**Figure 6:**
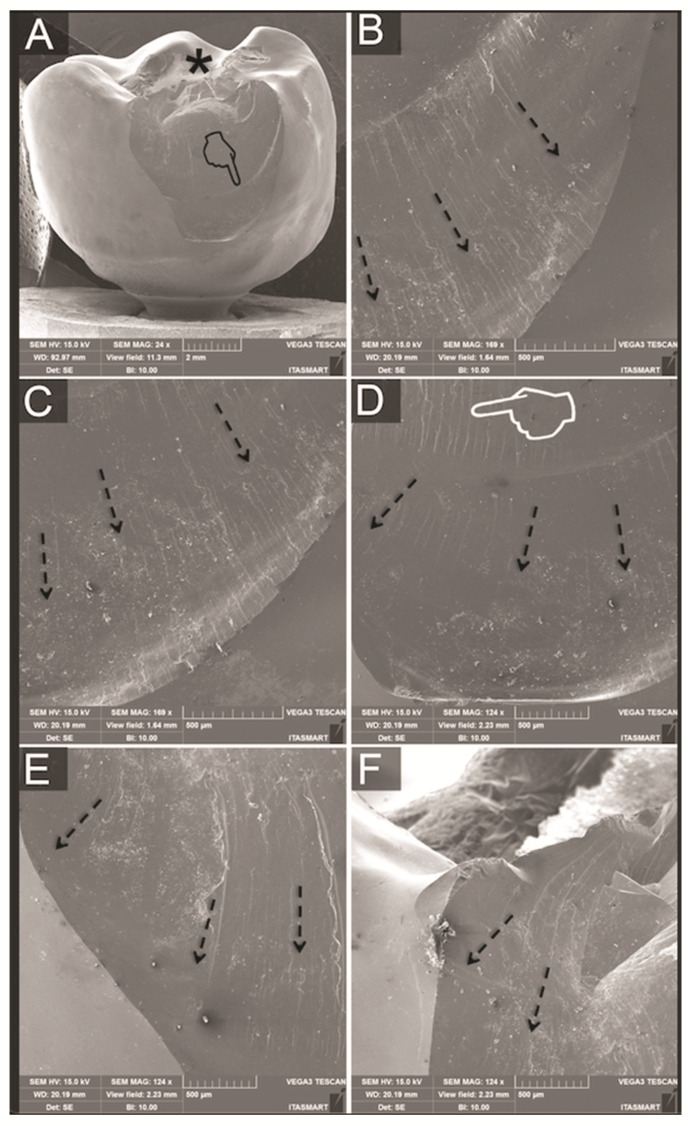
(**A**) Buccal view SEM micrograph of porcelain veneer cohesive fracture in a PFZ crown showing the indentation area (asterisk) and an arrest line (pointer) with its concave portion pointing towards the fracture origin. (**B**–**F**) are clockwise magnifications of crown margins showing direction of crack propagation towards the fractured margins, as indicated by hackle lines (dotted arrows) and by twist hackles (pointers).

**Figure 7:**
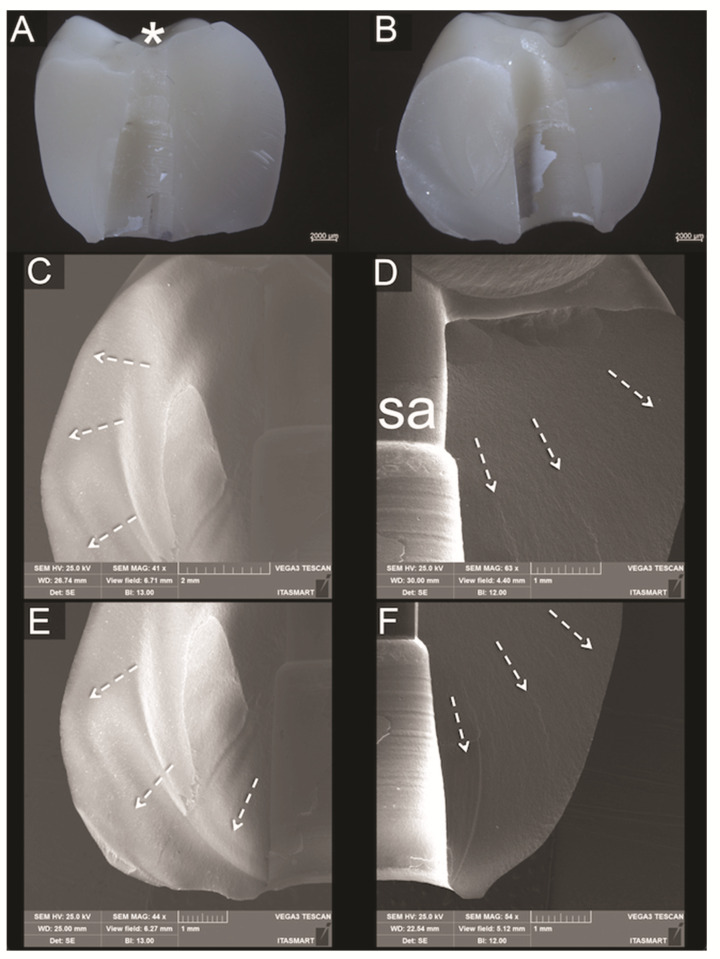
(**A**,**B**) are polarized light micrographs showing an overall view of the two matching fractured pieces of a monolithic lithium disilicate crown and the indentation region at the occlusal surface (asterisk). (**C**,**D**) are SEM occlusal magnifications of left and right sides of (**B**), respectively, both with screw access channel (sa) dividing the fracture and the direction of crack propagation indicated by dotted arrows. (**E**,**F**) are cervical magnifications of respective left and right views shown in B, which depict the crown intaglio surface area in contact with Ti-base and the direction of crack propagation indicated by dotted arrows.

No fractures of implants, screws or titanium bases could be observed for all groups after SLF. Analyses of the failed samples showed similar fractures for bi-layered crowns regarding overall fracture extension. However, whereas most PFM crowns resulted in porcelain veneer fractures exposing small parts of the metallic framework (Figure 5), failures in the PFZ crowns were mainly cohesive within the porcelain veneer (Figure 6). The fractured porcelain veneer surface of both groups left marks such as hackle lines, which allowed the detection of direction of fracture propagation. Arrest lines were observed with their concave portion pointing towards the fracture origin at the indentation area. In PFZ crowns, twist hackles, which are hackles that separate portions of the crack surface, each of which has rotated from the original crack plane in response to a lateral rotation or twist in the axis of principal tension, [25] were also observed. Fractures of monolithic lithium disilicate crowns (Figure 7) lead to bulk fracture splitting the crowns usually in two pieces where hackle lines were also observed showing the direction of crack propagation towards the fractured surface margins.

## 4. Discussion

This in vitro study investigated the fatigue behavior and failure load of different types of screw-retained hybrid abutment crowns. The tested null hypothesis was partially rejected as fatigue application did not have a significant effect on the investigated materials. However, significant effects between restoration materials could be determined.

All specimens of Group PFM and LDS survived dynamic loading up to 1.2 million cycles with simultaneous thermocycling. On the contrary, 16.67% of Group PFZ failed during chewing simulation due to major chipping fractures. These findings are in line with other in vitro studies on screw-retained PFM and all-ceramic ISSC. PFZ implant crowns showed more pronounced chipping fractures compared to metal-ceramic implant restorations during artificial aging [26]. However, other studies with chewing simulations of 1.2 million cycles at 100 N [27], observed no chipping events for hand-layered PFZ ISSC during fatigue.

In order to minimize the risk of chipping, it is necessary to ensure an anatomical, i.e., cusp-supporting framework design [28,29]. Very thick, very thin or irregularly applied veneering thicknesses may result in a lower survival or a higher complication rate [29]. In the present study, a CAD/CAM produced anatomical, cusp-supporting framework design was selected, providing a uniform veneer thickness. Failure rates for tooth-supported zirconium dioxide-based molar crowns decreased when an anatomical framework design, press veneering technique and a slow cooling protocol were applied like in the present study [30]. However, surface flaws already present during manufacturing due to thermal gradients and tempering stress in the veneer could also be responsible for premature chipping fractures [29,30]. The absence of reinforcing leucite crystals in the veneer of PFZ has shown to reduce 50% of its fracture toughness compared to the veneer of PFM, where leucite is used for thermal expansion compatibility with the metal substructure [31]. Altogether, these multifactorial aspects may help explain why PFZ implant crowns appear more susceptible to chipping fractures than PFM ISSC in the long-term [3]. Yet, the mean failure loads (>1500 N) of all investigated groups exceeded physiological chewing forces of 200–900 kN in the posterior area [32,33,34]. Thus, all tested restoration materials can be used for posterior screw-retained hybrid abutment crowns in clinical application.

Recent reviews [1,3,35] and clinical studies [36,37] comparing both cemented and screw-retained implant-supported restorations could not reveal a favorable type of retention over the other. However, at present, particularly for single-unit restorations screw-retained restorations are in favor, as these show lesser biological complications than cemented solutions and allow easier retrievability in case of technical complications [1,35,38,39]. Therefore, this laboratory study solely focused on screw-retained implant crowns bonded to prefabricated titanium bases. The highest numeric failure loads could be detected for monolithic LDS ISSC without and with fatigue. Other studies which investigated screw-retained ISSC in a thermomechanical chewing simulator (1.2 million cycles, 50 N, 1.6 Hz, 5°C to 55 °C) also recorded the highest fracture loads for LDS (IPS e.max CAD 3070 ± 376 N) in comparison to lithium silicate ceramics (Celtra Duo 2302 ± 798 N), and resin-matrix-ceramics (Cerasmart 977 ± 129 N and Enamic 1750 ± 277 N) [40]. In addition, when different setups of veneered zirconia implant crowns and screw-retained monolithic LDS ISSC after fatigue application (100 N, 1.2 million cycles) and aging (5 to 55 °C) were compared, LDS revealed again the highest fracture loads (1049.9 ± 145 N) [27].

Nevertheless, the above-obtained failure loads (1049.9 ± 145 N) were lower [27] as the results of the present study (LDS1 2722 ± 497 N). Test specimens in the aforementioned study [27] were exposed to single load to failure with an angulation of 30° and a smaller indenter (4 mm), which distributes the force application over a smaller area and could thus tend to cause an earlier failure. As different fatigue protocols with a variety of load and cycle scenarios are reported in the literature, a direct comparison between studies is difficult.

Two in vitro studies, which investigated the failure load of bi-layer and monolithic zirconia based ISSC molar restorations, recorded comparable fracture loads to the present study for PFZ before (1960 N) [41] and after fatigue (1520 N, 1.2 million cycles, 49 N, 10 k thermal cycles) [42]. However, PFM (G-96h, Kuraray Noritake) implant crowns performed inferior compared to the present study, both before (1450 N) [41] and after thermomechanical loading (1530 kN) [42].

Failure analysis in this study showed complete bulk fractures for monolithic LDS ISSC. PFM and PFZ ISSC presented both predominately porcelain veneer fractures, with PFM crowns frequently exposing the metal framework, whereas mainly chipping confined within the porcelain veneer was observed in PFZ crowns. Furthermore, no fractures or bending occurred on titanium implants. Likewise, no screw-loosening and debonding were observed. Thus, the weakest link of bi-layered ISSC is confirmed to be the veneer, while both frameworks showed no fractures or cracks during SLF. These observations are in line with the aforementioned in vitro studies [27,42].

Prospective clinical cohort studies confirm these results and reported survival rates of 100% for screw-retained press fabricated LDS ISSC after one year [12] and monolithic LDS CAD hybrid abutment crowns after two years [13]. A retrospective clinical study compared monolithic LDS and zirconia hybrid abutment crowns manufactured in a complete digital workflow [14]. Both restoration materials showed a survival rate of 100% after three years. One out of 19 LDS restorations showed a minor chipping fracture, which could be polished and did not require the exchange of the restoration [14].

To achieve a durable bond and a high failure load between ISSC and titanium bases, air-particle abrasion with a moderate pressure and subsequent bonding with a composite cement is recommended [43,44]. However, this only applies to titanium bases that do not have microgrooves. For these titanium bases, air-particle abrasion is contraindicated and yielded in lower bond strength values [45,46]. In the present study, the prosthetic height of the used titanium base was 4.7 mm. According to the all-ceramic manufacturer’s recommendation, a minimum prosthetic height of 4 mm is needed to bond LDS ISSC safely to titanium inserts [47]. This minimum height is essential to ensure an adequate bonding surface, otherwise premature failures and fractures might occur. A laboratory study, which used short titanium inserts of 3 mm for LDS and zirconia hybrid abutment crowns, observed a considerably high number (43.8% of LDS and 18.8% of zirconia specimens) of early failures (fractures as well as debondings) during thermomechanical loading in a mouth-motion fatigue simulator [48].

Possible study limitations might be the in vitro design itself, as the obtained results cannot be directly extrapolated to clinical long-term behavior. Moreover, ISSC was not manufactured in a complete digital workflow, which could be addressed in future research.

Based on the findings of this laboratory study, screw-retained monolithic LDS hybrid abutment crowns present a reliable treatment option for posterior single-missing teeth. Future research could address the influence of different heights of titanium inserts with regard to failure load of monolithic LDS and zirconia ISSC. Moreover, besides mechanical analysis, further studies should focus on biological reactions of the peri-implant interface of hybrid abutment crowns.

## 5. Conclusions

All investigated types of screw-retained hybrid abutment crowns showed failure loads above physiological forces in the posterior dentition. However, bi-layer porcelain-pressed-to-zirconia implant crowns are susceptible to premature chipping fractures. Within the limitations of this in vitro study, monolithic lithium disilicate ISSCs showed high failure loads and seem therefore a preferable restoration material for posterior screw-retained hybrid abutment crowns.

## Figures and Tables

**Table 1:** Descriptive statistics of failure loads in Newton (N) and standard deviation. Statistically significant differences (*p* < 0.05) are indicated by different superscript letters within a column without (small letter: a,b,c) and with (capital letter: A,B) fatigue exposure. *p*-values of t-test for influence of fatigue (*p* < 0.05).

Group	Without Fatigue	With Fatigue	Influence of Fatigue
	Mean ± SD	Mean ± SD	*p*-Value
LDS	2981 ± 798 ^a,b^	2722 ± 497 ^A^	*p* = 0.349
PFZ	2152 ± 572 ^c^	1686 ± 691 ^B^	*p* = 0.099
PFM	2633 ± 389 ^b,c^	2349 ± 578 ^A^	*p* = 0.099

## Data Availability

The data presented in this study are available on request from the corresponding author.
